# Validation of the Charlson Comorbidity Index for the prediction of 30-day and 1-year mortality among patients who underwent hip fracture surgery

**DOI:** 10.1186/s13741-024-00417-4

**Published:** 2024-07-03

**Authors:** Eveline de Haan, Benthe van Oosten, Veronique. A. J. I. M. van Rijckevorsel, T. Martijn Kuijper, Louis de Jong, Gert R. Roukema

**Affiliations:** 1 Surgery Department, Maasstad Hospital, Rotterdam, 3007 AC the Netherlands; 2 Surgery Department, Franciscus Hospital, Rotterdam, 3045 PM the Netherlands; 3 Maasstad Academy, Maasstad Hospital, Rotterdam, 3079 DZ the Netherlands

## Abstract

**Introduction:**

The aim of our study was to validate the original Charlson Comorbidity Index (1987) (CCI) and adjusted CCI (2011) as a prediction model for 30-day and 1-year mortality after hip fracture surgery. The secondary aim of this study was to verify each variable of the CCI as a factor associated with 30-day and 1-year mortality.

**Methods:**

A prospective database of two-level II trauma teaching hospitals in the Netherlands was used. The original CCI from 1987 and the adjusted CCI were calculated based on medical history. To validate the original CCI and the adjusted CCI, the CCI was plotted against the observed 30-day and 1-year mortality, and the area under the curve (AUC) was calculated.

**Results:**

A total of 3523 patients were included in this cohort study. The mean of the original CCI in this cohort was 5.1 (SD ± 2.0) and 4.6 (SD ± 1.9) for the adjusted CCI. The AUCs of the prediction models were 0.674 and 0.696 for 30-day mortality for the original and adjusted CCIs, respectively. The AUCs for 1-year mortality were 0.705 and 0.717 for the original and adjusted CCIs, respectively.

**Conclusions:**

A higher original and adjusted CCI is associated with a higher mortality rate. The AUC was relatively low for 30-day and 1-year mortality for both the original and adjusted CCIs compared to other prediction models for hip fracture patients in our cohort. The CCI is not recommended for the prediction of 30-day and 1-year mortality in hip fracture patients.

**Supplementary Information:**

The online version contains supplementary material available at 10.1186/s13741-024-00417-4.

## Introduction

As life expectancy is rising globally, the incidence of hip fractures is increasing (Man et al. 2016). Surgical treatment is recommended for most hip fractures, aiming for pain relief and early mobilization (Jameson et al. 2012; Nichols et al. 2017; Bhandari and Swiontkowski 2017). Despite therapy, hip fractures are associated with high mortality rates: 30-day and 1-year mortality rates have been reported between 6.4–13.3% and 23.2–33.0%, respectively (Hu et al. 2012; Dubljanin Raspopovic et al. 2014; Barceló et al. 2021; Gundel et al. 2020; Roche et al. 2005). Mortality after hip surgery strongly depends on comorbidities and perioperative factors (Hu et al. 2012; Barceló et al. 2021; Gundel et al. 2020; Roche et al. 2005). Accurate preoperative assessment of mortality risk after hip fracture surgery can improve perioperative management and will be helpful for guiding clinical decision-making and appropriate informed consent.

**Fig. 1:**
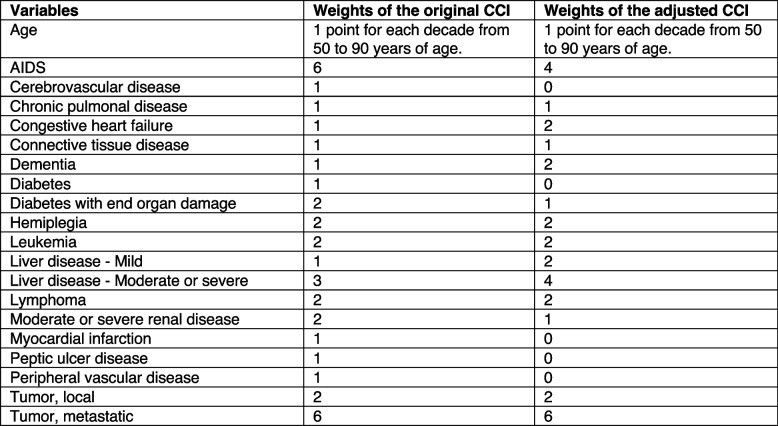
The variables and weights of the original and adjusted CCIs (Charlson et al. 1987; Quan et al. 2011)

Preoperative risk factors for mortality have been identified (Hu et al. 2012; Barceló et al. 2021; Gundel et al. 2020; Roche et al. 2005; Dodd et al. 2016; Smith et al. 2014), and various risk models assessing patients’ risk of mortality have been developed (Ramanathan et al. 2005; Maxwell et al. 2008; Charlson et al. 1987; Ree et al. 2020). A commonly used assessment scale for predicting 1-year mortality in the general population is the Charlson Comorbidity Index (CCI) (Charlson et al. 1987). The CCI is used to predict 1-year mortality by classifying age and 19 comorbidities, each assigned a score of 1, 2, 3, or 6 (Fig. 1) (Charlson et al. 1987). The cumulative score results in the CCI. The 1-year mortality rates associated with CCI scores were as follows: CCI = 0.12%; CCI = 1–2.26%; CCI = 3–4.52%; CCI > 5 85% (Charlson et al. 1987). The original CCI was published in 1987 (Charlson et al. 1987). In 1994, the original CCI was validated by the developers of the original CCI (Charlson et al. 1994). In 2011, the CCI was updated and validated. The revision was based on changes in the contribution of comorbidities since 1987, resulting in an adjustment to the weight of several comorbidities (Fig. 1) (Quan et al. 2011).

The CCI has recently been validated for multiple diseases separately (Zhou et al. 2022; Radovanovic et al. 2014; Birim et al. 2003). The original and adjusted CCI has limited validation in patients with a hip fracture for 30-day and 1-year mortality (Haugan et al. 2021; Karres et al. 2015). The AUCs of these models were lower than those of other prediction models for hip fracture patients (Maxwell et al. 2008; Ree et al. 2020). Not all validation conditions and statistics were met during previous validation of the CCI for 30-day and 1-year mortality in hip fracture patients. Furthermore, there is no complete overview of the performance of the original and adjusted CCI for both 30-day and 1-year mortality prediction after hip fracture surgery. In conclusion, there is currently no consensus regarding the utilization of the CCI in clinical practice.

The aim of our study was to validate the original CCI and adjusted CCI from 2011 as risk prediction models for 30-day and 1-year mortality after hip fracture surgery. The secondary aim of this study was to verify that each variable of the CCI is associated with 30-day and 1-year mortality.

## Methods

A prospective database of two-level II trauma teaching hospitals in the Netherlands was used. We prospectively included all patients (*n* = 3523) who underwent hemiarthroplasty between 2011 and 2016 and all patients who underwent hip fracture surgery between 2018 and 2021. Patients who underwent hip fracture surgery based on a pathological fracture, a periprosthetic fracture, or patients who underwent a primary Girdelstone operation based on a palliative trajectory were excluded.

Baseline characteristics and clinical outcomes were retrospectively obtained from the hospital records. Only comorbidities who were diagnosed prior to surgery were included. The follow-up time after surgery was at least 1 year. The mortality data were obtained by verifying the number of citizen services provided to the corresponding municipality.

The original CCI developed in 1987 and the adjusted CCI from 2011 were calculated based on medical history. The original CCI was calculated based on age and 17 comorbidities (Fig. 1) (Charlson et al. 1987). The age-adjusted CCI from 2011 was calculated based on age and 12 comorbidities (Fig. 1) (Quan et al. 2011). All comorbidities were weighted between 1 and 6. Age was weighted as 1 point for each decade from 50 to 90 years of age for both the original CCI and the adjusted CCI from 2011. The cumulative weight results in the CCI.

Diabetes with chronic complications was difficult to assess. The primary etiology (for example, diabetes) of nephropathy, neuropathy, or retinopathy is frequently not completely clear. Therefore, patients in our cohort were only classified as diabetic patients without chronic complications. Distinguishing between mild liver disorders and moderate or severe liver disease is based on the presence of portal hypertension and variceal bleeding and may not be well reported in medical charts. If the severity of the liver disorder was unclear, patients were categorized into the mild liver disorder group.

### Statistical analysis

Categorical variables are presented as frequencies and percentages. Continuous variables are presented as the mean (standard deviation, ± SD) if they were normally distributed. The associations between the variables of the CCI and 30-day and 1-year mortality after hip fracture surgery were tested by univariate analysis. All regression analyses were two-sided with a significance level of *p* < 0.05. The results are presented as odds ratios (ORs) with 95% confidence intervals (CIs). We assessed 30-day and 1-year mortality rates for each level of the original and adjusted CCI.

The CCI is a score does not provide a predicted mortality. To translate this risk score into a predicted mortality we fitted a complementary log–log model to our dataset. The calculated CCI as a single linear predictor was used to obtain the predicted mortality.

To validate the original CCI and the adjusted CCI from 2011, we plotted the CCI against the observed 30-day and 1-year mortality and calculated the area under the curve (AUC) of the receiver operating characteristic curve (ROC). Calibration plots were generated by classifying patients according to their predicted mortality (estimated by a generalized model using a log–log link) in ten equally sized clusters, plotting for each cluster the percentage of observed mortality against the mean predicted mortality. Statistical analyses were performed using Stata version 14.0 (StataCorp, College Station, TX, USA).

### Ethics

The study protocol was approved by the local ethics committee (L2017044, Toetsingscommissie Wetenschappelijk Onderzoek Rotterdam (TWOR), Rotterdam, Trial registration number NL8313) on 14 February 2020. Due to the absence of any changes in the standard practice of care and a high percentage of cognitive dysfunction among the patients, the local ethics committee determined that patients’ consent to review their medical records was not needed. All patient data were collected anonymously, and all protocols were conducted in compliance with the Declaration of Helsinki. No external funding was used for this study.

## Results

**Fig. 2:**
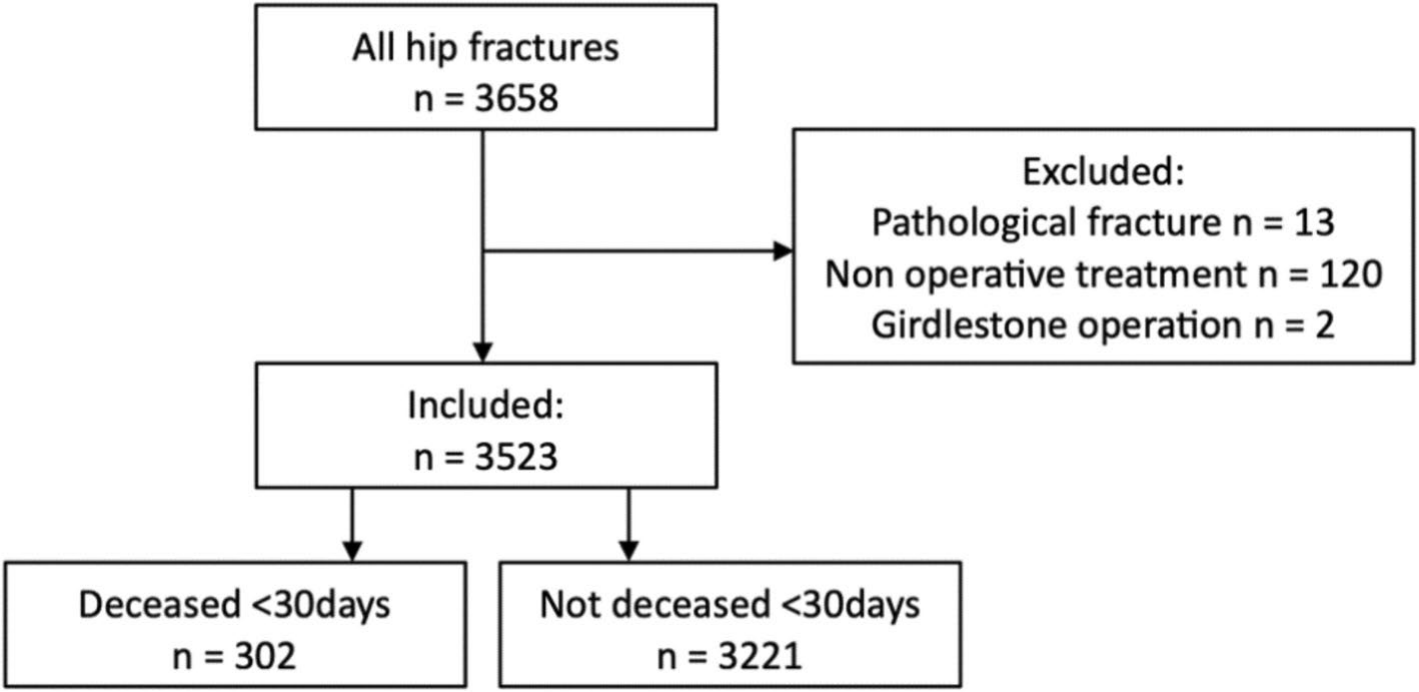
Flow chart of included patients

A total of 3523 patients were included in this cohort study (Fig. 2). The mean of the original CCI in this cohort was 5.1 (SD ± 2.0) and 4.6 (SD ± 1.9) for the adjusted CCI. A total of 8.6% of all patients died within 30 days after surgery. The 1-year mortality was 25.9%. The median follow-up of the patients who did not die within 1 year after surgery was 366 days (IQR 365–465).

### Variables of the CCI

The associations between the variables of the CCI and 30-day and 1-year mortality after hip fracture surgery were tested by univariate analysis (Table 1). Age above 71 years was associated with higher 30-day mortality, and age above 61 years was associated with higher 1-year mortality. Congestive heart failure, dementia, diabetes, and myocardial infarction (MI) (4 out of 17 variables) were significantly associated with increased 30-day mortality. Cerebrovascular disease, congestive heart failure, dementia, diabetes, leukemia, moderate or severe renal disease, myocardial infarction, and any tumor (local or metastatic) were associated with increased 1-year mortality (8 out of 17 variables). The original and adjusted CCIs were significantly higher in the 30-day and 1-year mortality cohorts compared to the cohort who had not died at that time point.

**Table 1:** Univariable analyses of risk and prognostic baseline factors

Variable	All patients (3523)	Deceased < 30 days (302)	Not deceased < 30 days (3221)	Odds ratio	*P*-value	Deceased < 1 year (911)	Not deceased < 1 year (2612)	Odds ratio	*P*-value
	n (%)	n (%)	n (%)	(95% CI)		n (%)	n (%)	(95% CI)	
**Age**									
< 50	71 (2.0%)	0 (0.0%)	71 (2.2%)	-	-	0 (0.0%)	71 (2.7%)	-	-
60–70 (ref.)	121 (3.4%)	1 (0.3%)	120 (3.7%)	-	**-**	4 (0.4%)	117 (4.5%)	-	-
61–70	335 (9.5%)	10 (3.3%)	325 (10.1%)	3.69 (0.47 – 29.15)	0.215	37 (4.1%)	298 (11.4%)	3.63 (1.27 – 10.41)	**0.016**
71–80	825 (23.4%)	48 (15.9%)	777 (24.1%)	7.41 (1.01 – 54.21)	**0.048**	151 (16.6%)	674 (25.8%)	6.55 (2.38 – 18.03)	** < 0.001**
81–90	1519 (43.1%)	137 (45.4%)	1382 (42.9%)	11.90 (1.65 – 85.81)	**0.014**	438 (48.1%)	1081 (41.4%)	11.85 (4.35 – 32.30)	** < 0.001**
> 91	652 (18.5%)	106 (35.1%)	546 (17.0%)	23.30 (3.22 – 168.58)	**0.002**	281 (30.8%)	371 (14.2%)	22.15 (8.08 – 60.74)	** < 0.001**
**Variables of the CCI**									
AIDS	4 (0.1%)	1 (0.3%)	3 (0.1%)	1.24 (0.85 – 1.80)	0.272	1 (0.1%)	3 (0.1%)	0.99 (0.68 – 1.45)	0.969
Cerebrovascular disease	721 (20.5%)	73 (24.2%)	648 (20.1%)	1.27 (0.96 – 1.67)	0.096	235 (25.8%)	486 (18.6%)	1.52 (1.27 – 1.82)	** < 0.001**
Chronic pulmonal disease	316 (9.0%)	34 (11.3%)	282 (8.8%)	1.32 (0.91 – 1.93)	0.147	93 (10.2%)	223 (8.5%)	1.22 (0.94 – 1.57)	0.129
Congestive heart failure	675 (19.2%)	77 (25.5%)	598 (18.6%)	1.50 (1.14 – 1.97)	**0.004**	231 (25.4%)	444 (17.0%)	1.66 (1.38 – 1.99)	** < 0.001**
Connective tissue disease	156 (4.4%)	9 (3.0%)	147 (4.6%)	0.64 (0.32 – 1.27)	0.204	33 (3.6%)	123 (4.7%)	0.76 (0.51 – 1.13)	0.171
Dementia	804 (22.8%)	137 (45.4%)	667 (20.7%)	3.18 (2.50 – 4.05)	** < 0.001**	354 (38.9%)	450 (17.2%)	3.05 (2.58 – 3.61)	** < 0.001**
Diabetes	644 (18.3%)	71 (23.5%)	573 (17.8%)	1.42 (1.07 – 1.88)	**0.014**	219 (24.0%)	425 (16.3%)	1.63 (1.35 – 1.96)	** < 0.001**
Diabetes with end organ damage	0 (0.0%)	-	-	-	-	-	-	-	-
Hemiplegia	121 (3.4%)	11 (3.6%)	110 (3.4%)	1.03 (0.75 – 1.42)	0.836	40 (4.4%)	81 (3.1%)	1.20 (0.99 – 1.45)	0.067
Leukemia	14 (0.4%)	1 (0.3%)	13 (0.4%)	0.91 (0.33 – 2.51)	0.848	7 (0.8%)	7 (0.3%)	1.70 (1.00 – 2.87)	**0.048**
Liver disease									
Mild	10 (0.3%)	1 (0.3%)	9 (0.3%)	1.19 (0.15 – 9.39)	0.872	2 (0.2%)	8 (0.3%)	0.72 (0.15 – 3.38)	0.674
Moderate or severe	13 (0.4%)	1 (0.3%)	12 (0.4%)	0.89 (0.12 – 6.86)	0.910	4 (0.4%)	9 (0.3%)	1.27 (0.39 – 4.15)	0.687
Lymphoma	25 (0.7%)	0 (0.0%)	25 (0.8%)	-	-	4 (0.4%)	21 (0.8%)	0.74 (0.43 – 1.26)	0.266
Moderate or severe renal disease	59 (1.7%)	9 (3.0%)	50 (1.6%)	1.40 (0.97 – 2.00)	0.069	23 (2.5%)	36 (1.4%)	1.36 (1.05 – 1.77)	**0.022**
Myocardial infarction	320 (9%)	37 (12.3%)	283 (8.8%)	1.45 (1.01 – 2.09)	**0.046**	109 (12.0%)	211 (8.1%)	1.55 (1.21 – 1.98)	** < 0.001**
Peptic ulcer disease	3 (0.1%)	1 (0.3%)	2 (0.1%)	5.35 (0.48 – 59.14)	0.172	1 (0.1%)	2 (0.1%)	1.43 (0.13 – 15.8)	0.769
Peripheral vascular disease	122 (3.5%)	13 (4.3%)	109 (3.4%)	1.28 (0.71 – 2.31)	0.404	36 (4.0%)	86 (3.3%)	1.21 (0.81 – 1.80)	0.349
Tumor									
Local	412 (11.7%)	38 (12.6%)	374 (11.6%)	1.09 (0.77 – 1.56)	0.621	16 (1.8%)	266 (10.2%)	1.70 (1.36 – 2.11)	** < 0.001**
Metastatic	16 (0.5%)	1 (0.3%)	15 (0.5%)	0.72 (0.09 – 5.46)	0.749	8 (0.9%)	8 (0.3%)	3.09 (1.16 – 8.26)	**0.025**
**Original CCI** (mean ± SD)	5.05 (± 1.99)	6.05 (± 1.61)	4.95 (± 2.00)	1.30 (1.23 – 1.38)	** < 0.001**	6.05 (± 1.69)	4.70 (± 1.97)	1.45 (1.39 – 1.51)	** < 0.001**
**Adjusted CCI** (mean ± SD)	4.64 (± 1.85)	5.76 (± 1.61)	4.54 (± 1.84)	1.42 (1.33 – 1.51)	** < 0.001**	5.65 (± 1.59)	4.29 (± 1.81)	1.55 (1.48 – 1.63)	** < 0.001**

*CCI* Charlson Comorbidity Index, *AIDS* acquired immunodeficiency syndrome

### The CCI and mortality rate

**Fig. 3:**
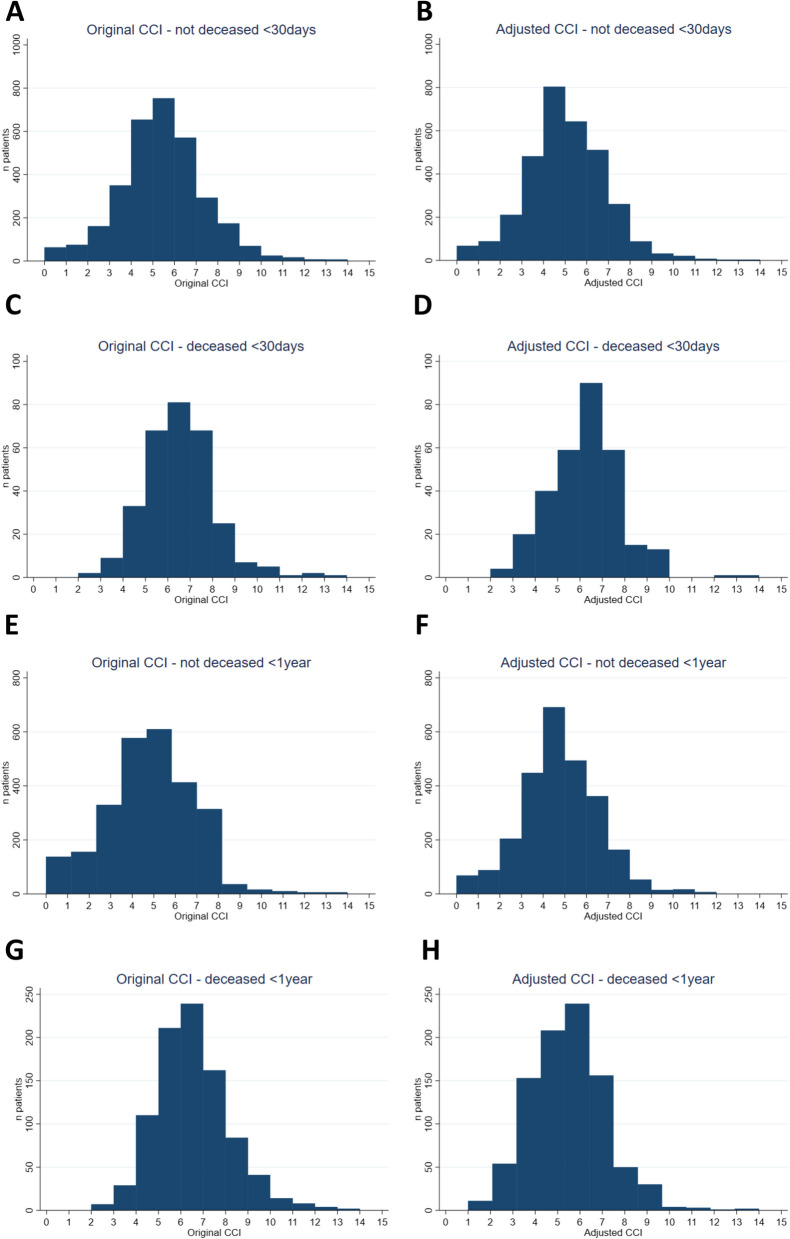
The original and adjusted CCIs for the whole cohort and for patients who died within 30 days and 1 year after hip fracture surgery. **A** The original CCI for patients who did not die within 30 days after surgery. **B** The adjusted CCI for patients who did not die within 30 days after surgery. **C** The original CCI for patients who died within 30 days after surgery. **D** The adjusted CCI for patients who died within 30 days after surgery. **E** The original CCI for patients who did not die within 1 year after surgery. **F** The adjusted CCI for patients who did not die within 1 year after surgery; **G** The original CCI for patients who died within 1 year after surgery. **H** The adjusted CCI for patients who died within 1 year after surgery

The distributions of the original and adjusted CCIs of all patients and patients who died within 30 days and 1 year after hip fracture surgery are visualized in histograms (Fig. 3). The percentages of observed mortality for each score of the original CCI and the adjusted CCI are shown in Table 2.

**Table 2:** Mortality rates for the original and adjusted CCI in this cohort

**Value of the CCI**	**Original CCI**		**Adjusted CCI**	
	**30-day mortality**	**1-year mortality**	**30-day mortality**	**1-year mortality**
0	0 / 63 (0%)	0 / 63 (0%)	0 / 68 (0%)	0 / 68 (0%)
1	0 / 75 (0%)	0 / 75 (0%)	0 / 89 (0%)	1 / 89 (1%)
2	2 / 163 (1%)	7 / 163 (4%)	4 / 215 (2%)	10 / 215 (5%)
3	9 / 359 (3%)	29 / 359 (8%)	20 / 502 (4%)	54 / 502 (11%)
4	33 / 687 (5%)	110 / 687 (16%)	40 / 844 (5%)	153 / 844 (18%)
5	68 / 821 (8%)	211 / 821 (26%)	59 / 702 (8%)	208 / 702 (30%)
6	81 / 652 (12%)	239 / 652 (37%)	90 / 601 (15%)	239 / 601 (40%)
7	68 / 361 (19%)	162 / 361 (45%)	59 / 320 (18%)	156 / 320 (49%)
8	25 / 199 (13%)	84 / 199 (42%)	15 / 103 (15%)	50 / 103 (49%)
9	7 / 77 (9%)	41 / 77 (53%)	13 / 45 (29%)	30 / 45 (67%)
10	5 / 30 (17%)	14 / 30 (47%)	0 / 21 (0%)	4 / 21 (19%)
11	1 / 18 (6%)	8 / 18 (44%)	0 / 8 (0%)	3 / 8 (38%)
12	2 / 10 (20%)	4 / 10 (40%)	1 / 3 (33%)	1 / 3 (33%)
13	0 / 3 (0%)	0 / 3 (0%)	0 / 0 (-)	0 / 0 (-)
14	1 / 5 (20%)	2 / 5 (40%)	1 / 2 (50%)	2 / 2 (100%)
**Total**	302 / 3523 (9%)	911 / 3523 (26%)	302 / 3523 (9%)	911 / 3523 (26%)
0	0 / 63 (0%)	0 / 63 (0%)	0 / 68 (0%)	0 / 68 (0%)
1–2	2 / 238 (1%)	7 / 238 (3%)	4 / 304 (1%)	11 / 304 (4%)
3–4	42 / 1046 (4%)	139 / 1046 (13%)	60 / 1346 (4%)	207 / 1346 (15%)
≥ 5	258 / 2176 (12%)	765 / 2176 (35%)	238 / 1805 (13%)	693 / 1805 (38%)

There is minimal variability in the amount of the original CCI and the adjusted CCI (for both 30-day and 1-year mortality) up to a value of 8. From a CCI of 9 or higher, there will be more variability in the distribution. The variability can be explained by the scarcity of patients with a CCI equal to or higher than 9 in this study cohort.

The original CCI of the patients who died within 30 days after surgery was in 85% of the patients higher or equal to five. This percentage was 79% for adjusted CCI for 30-day mortality. When analyzing the 1-year mortality, an original CCI of 5 or higher was found in 84% of the deceased patients and the adjusted CCI in 76% of the deceased patients.

### Performance and validation of the CCI

**Fig. 4:**
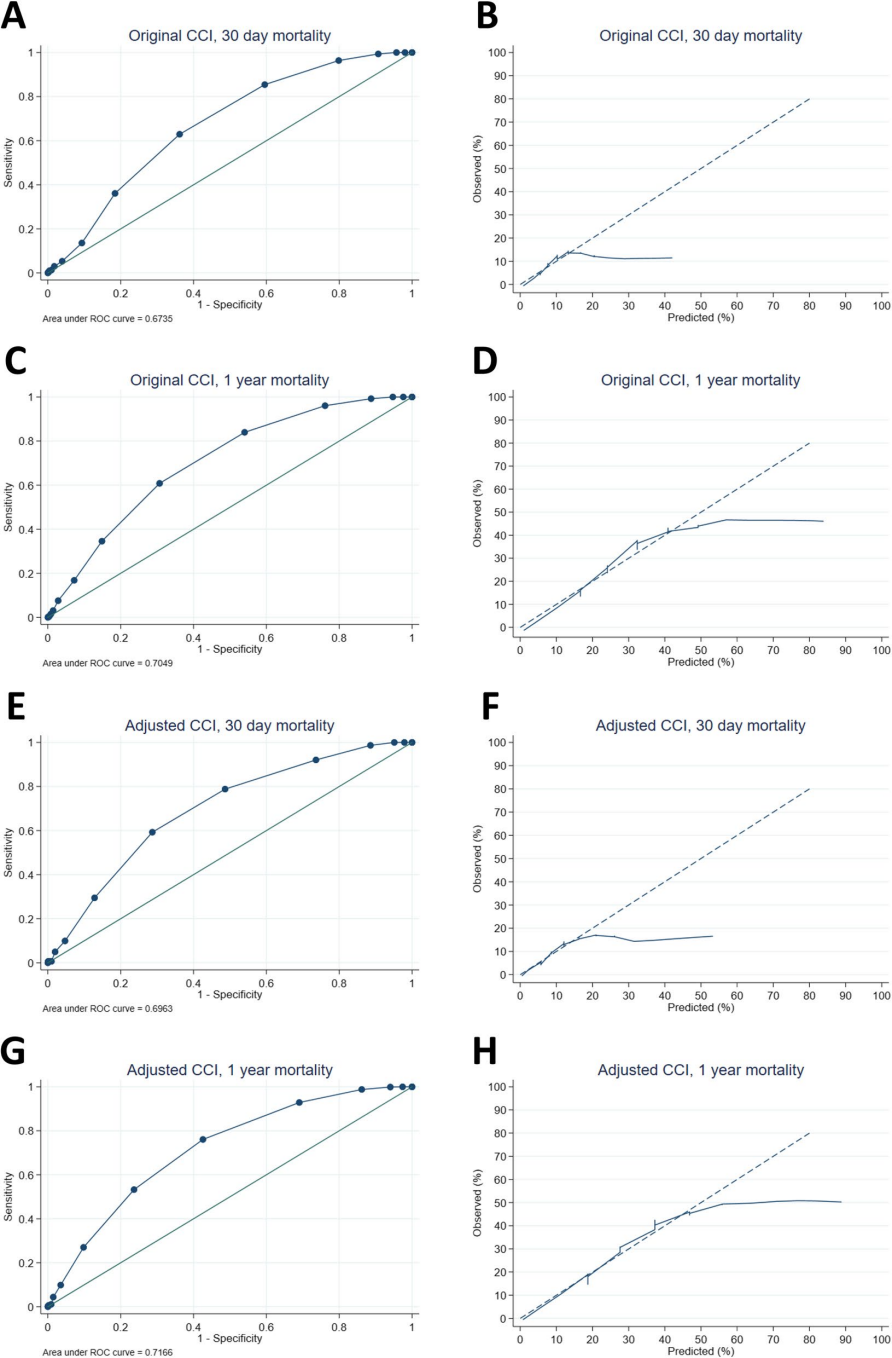
Receiver operating characteristic (ROC) curves (**A**, **C**, **E**, and **G**) and calibration plots (**B**, **D**, **F**, and H) of the original CCI (**A**, **B**, **C**, **D**) and adjusted CCI (**E**, **F**, **G**, and **H**) for 30-day mortality (**A**, **B**, **E**, and **F**) and 1-year mortality (**C**, **D**, **G**, and **H**)

The performance statistics of the original and adjusted CCIs are shown in Fig. 4. The AUCs were 0.674 and 0.696 for 30-day mortality for the original and adjusted CCIs, respectively. The AUCs for 1-year mortality were 0.705 and 0.717 for the original and adjusted CCIs, respectively. The 95% CIs of the AUCs are shown in Appendix 1.

## Discussion

A commonly used assessment scale to predict 1-year mortality in the general population is the CCI. (Charlson et al. 1987). However, it is unclear whether the CCIs predicted 1-year mortality aligns with the actual clinical outcomes of patients with hip fractures. Based on the high 30-day mortality rate within the hip fracture population (Hu et al. 2012; Barceló et al. 2021; Gundel et al. 2020; Roche et al. 2005; Dubljanin Raspopovic et al. 2015), it is also helpful to know whether a CCI is related to a higher 30-day mortality. The aim of our study was to validate the original CCI and adjusted CCI from 2011 as risk prediction models for 30-day and 1-year mortality after hip fracture surgery.

The mean of the original CCI in this cohort was 5.1 (SD ± 2.0) and 4.6 (SD ± 1.9) for the adjusted CCI. A previous study of a hip fracture population reported a similar CCI value of 5 (SD ± 2) (Shen et al. 2022). The observed 30-day and 1-year mortality rates in this study were 8.6% and 25.9%, respectively. This is in accordance with previously reported 30-day mortality rates between 6.4% and 13.3% and 1-year mortality rates between 23.2% and 33.0% (Hu et al. 2012; Dubljanin Raspopovic et al. 2014; Barceló et al. 2021; Gundel et al. 2020; Roche et al. 2005).

### Variables of the CCI

Based on the univariate analysis, patients older than 71 years were associated with a higher 30-day mortality rate, and patients with an age above 61 years were associated with a higher 1-year mortality. Increasing age is a well-established risk factor for both 30-day and 1-year mortality after hip fracture surgery (Hu et al. 2012; Barceló et al. 2021; Gundel et al. 2020; Roche et al. 2005; Huette et al. 2020). It seems justifiable that the CCI increases with age.

Based on our study results and a recently published meta-analysis, the medical history of dementia had a substantial influence on mortality after hip fracture (Bai et al. 2018). It appears reasonable that the weight assigned to dementia in the adjusted CCI has been increased from 1 to 2. The 30-day and 1-year mortality rates were significantly higher in patients with diabetes, which is in line with previous literature (Frenkel Rutenberg et al. 2021). Nevertheless, the weight assigned to diabetes was decreased after adjustment. Congestive heart failure has been described in the literature as a risk factor for in-hospital and 1-year mortality (Sanz-Reig et al. 2017; Cenzer et al. 2016; Guzon-Illescas et al. 2019), which is in line with our univariable analysis results. In our cohort, MI was associated with higher 30-day and 1-year mortality. MI is not described in the literature in relation to mortality in hip fracture patients. Nevertheless, Guzon-Illescas et al. (2019) also reported a relationship between higher overall mortality and MI in medical history (Guzon-Illescas et al. 2019). The fact that MI is no longer included in the adjusted CCI is debatable for hip fracture patients. MI can be an ambiguous variable since a history of MI does not provide any information on the remaining heart function, which may be more relevant. A medical history of cerebrovascular disease was associated with increased 1-year mortality in this study. However, previous studies have reported no correlation between prior cerebrovascular accidents and in-hospital or 1-year mortality after hip fracture surgery (Sanz-Reig et al. 2017; Guzon-Illescas et al. 2019; Youm et al. 2000). It is questionable that cerebrovascular accidents are no longer included in the adjusted CCI for hip fracture patients.

Moderate or severe renal disease was significantly associated with a higher 1-year mortality rate. There were no significant differences in mortality among patients with mild, moderate, or severe liver disease in our cohort. This is probably due to the small number of patients in our cohort. Patients with local tumors, metastatic tumors, and leukemia had significantly higher 1-year mortality. Lymphoma and acquired immunodeficiency syndrome (AIDS) were not significantly associated with 30-day or 1-year mortality after univariable analyses.

Chronic pulmonary disease, connective tissue disease, hemiplegia, peripheral vascular disease, and peptic ulcer disease were not associated with increased mortality in our cohort. These factors have not been described in the literature as risk factors for mortality among hip fracture patients. A higher CCI is well described in the literature as a risk factor for both 30-day and 1-year mortality (Gundel et al. 2020; Smith et al. 2014; Borge et al. 2022; Forssten et al. 2021. This finding is in line with our univariable analysis results for both the original and adjusted CCIs.

### The CCI and mortality rates

On average, the original CCI was approximately 1 point higher in the 30-day mortality and 1-year mortality groups than in patients who did not die (Fig. 3). The adjusted CCI showed a slightly larger difference between the entire cohort for 30-day mortality of approximately 2 points but also appeared to be 1 point greater for 1-year mortality than in patients who did not die. Ideally, by predicting mortality, the differences between the patients who died and those who did not die are more clearly reflected in the histogram (Fig. 3).

The percentages of observed mortality for each score of the original CCI and the adjusted CCI are shown in Table 2. The mortality rate continued to increase to approximately 9 points on the CCI. After a CCI of 9 points, a decrease in the mortality rate can be perceived, possibly because of the small number of patients included in these categories. The observed 1-year mortality rates of the original CCI scores are described in the article by M. Charlson: CCI 0 = 12%; CCI 1–2 = 26%; CCI 3–4 = 52%; and CCI 5 = 85% (Charlson et al. 1987). In our cohort, these percentages were 0%, 3%, 13%, and 35%, respectively, for the original CCI and 0%, 4%, 15%, and 38%, respectively, for the adjusted CCI (Table 2). The 1-year mortality percentages from 1984 were not comparable to the 1-year mortality percentage from this cohort for either the original CCI or the adjusted CCI.

### Performance and validation of the CCI

The AUCs were 0.674 for the original CCI and 0.696 for the adjusted CCI for 30-day mortality. The AUCs for 1-year mortality were 0.705 and 0.717 for the original and adjusted CCIs, respectively (Fig. 4). Tang et al. (2021) reported an AUC for the CCI of 0.653 for in-hospital mortality (Tang et al. 2021). The adjusted CCI was previously validated by Haugan et al. (2021), who reported an AUC of 0.726 for 30-day mortality and 0.751 for 1-year mortality (Haugan et al. 2021). However, Haugan et al. (2021) did not report mortality rates, studied each variable of the CCI, generated calibration plots, and did not validate the original CCI. Karres et al. (2015) validated 6 prediction models for 30-day mortality after hip fracture surgery and found an AUC of 0.72 for the original CCI (Karres et al. 2015).

The calibration curves for 30-day mortality for both the original and adjusted CCIs demonstrated inadequate alignment between the CCI and observed 30-day mortality. The CCI did not differ between the patients with approximately 20% predicted mortality or higher, and the same mortality was observed for all those patients. Therefore, there is overprediction from approximately 20% of the predicted mortality. The calibration curve for 1-year mortality showed improved alignment for both the original and adjusted CCI, but the curve also strongly deflects from the 40% observed mortality for both the original and adjusted CCI.

Other prediction models for 30-day and 1-year mortality for hip fracture patients have recently been developed (Maxwell et al. 2008; Ree et al. 2020). The Nottingham Hip Fracture Score (NHFS) was validated by Sun et al. (2023) and reached an AUC of 0.791 for 30-day mortality (Sun et al. 2023). The Brabant Hip Fracture Score (BHFS) was internally validated and had AUCs of 0.71 and 0.75 for 30-day and 1-year mortality, respectively (Ree et al. 2020). Both the NHFS and the BHFS show better performance statistics than the CCI. The NHFS and BHFS do allow direct calculation of the mortality risk for a specific patient; hence, a certain CCI score does not directly translate into a predicted 30-day or 1-year mortality rate.

Based on the above findings, we do not recommend the use of the CCI as a prediction model for both 30-day and 1-year mortality after hip fracture surgery.

## Strengths

In this study, we investigated a large cohort of hip fracture patients with detailed descriptions of baseline and perioperative factors in a prospective hip fracture database. The cohorts are representative of the target population and have limited missing data (including limited missing data for the CCI variables), increasing the external validity of the findings. The data on the primary outcome measures, 30-day mortality, and 1-year mortality, were complete. The sample size and particularly the number of events of the test cohort included more than 100 patients, and almost all baseline factors (including the variables of the CCI) were complete, indicating that adequate validation could be conducted (Ramspek et al. 2021). Extensive external validation was performed, including both statistical and graphical assessments of the discrimination and calibration, which made the results more reliable than those of previous studies. This is the first study in which the use of the CCI is not recommended for the prediction of 30-day and 1-year mortality in hip fracture patients, as better prediction models are currently available.

## Limitations

This was an observational cohort study based on patients’ medical charts, meaning that potentially unreported data were not included in our analysis. Unreported comorbidities were not included in the analyses, which is a major limitation of this study given the comorbidities were necessary to calculate the primary outcome (CCI). However, the comprehensive database encompasses all consecutive patients, and due to careful status research and follow-up, the amount of missing data was very limited. The CCI may be lower in this cohort than in the whole population because of missing data on diabetes with end-organ failure and the severity of liver disease. Nevertheless, this difference should not be large due to the relatively low incidence of these diseases in a broader population. Additionally, we excluded the most fragile patients who were treated nonoperatively because we aimed to investigate risk factors for 30-day mortality after hip fracture surgery.

## Conclusion

A complete overview of the performance of the original and adjusted CCI for both 30-day and 1-year mortality prediction after hip fracture surgery was established. A higher original or adjusted CCI is associated with higher mortality rates in hip fracture patients. Nevertheless, in this cohort, the AUC of the original and adjusted CCI for 30-day and 1-year mortality was approximately 0.70. This finding is in line with previous literature. More adequate prediction models for mortality after hip fracture are available. Moreover, the CCI does not formally provide prediction rules for 30-day and 1-year mortality in hip fracture patients. Hence, a certain CCI score does not directly translate into a predicted 30-day or 1-year mortality rate. Other prediction models allow the direct calculation of the mortality risk for a specific patient (Maxwell et al. 2008; Ree et al. 2020). The CCI is not recommended for the prediction of 30-day or 1-year mortality in hip fracture patients.

### Supplementary Information

Supplementary Material 1.

## Data Availability

No datasets were generated or analysed during the current study.On request.
