# Supplementary material for: Validation of the Charlson Comorbidity Index for the prediction of 30-day and 1-year mortality among patients who underwent hip fracture surgery
Source: Perioper Med (Lond). 2024 Jul 3;13:67. doi: 10.1186/s13741-024-00417-4 (PMC11223422; doi:10.1186/s13741-024-00417-4)
Supplement: Supplementary file 1 — Supplementary Material 1. [file 13741_2024_417_MOESM1_ESM.docx]

| **Appendix 1.** Discrimination data | | |  |  |
| --- | --- | --- | --- | --- |
|  | **Original CCI**  **30 days mortality** | **Original CCI**  **1 year mortality** | **Adjusted CCI**  **30 days mortality** | **Adjusted CCI**  **1 year mortality** |
| **Discrimination** |  |  |  |  |
| - AUC ROC | 0.674 (0.646 - 0.701) | 0.705 (0.687 - 0.723) | 0.696 (0.668 - 0.725) | 0.717 (0.699 - 0.735) |
| Abbreviations: AUC: Area under the Curve. ROC: Receiver Operating Characteristics | | | | |
|  | | | | |
